# Spinocerebellar Ataxia 27 A with Episodic Ataxia: Case Series of Fibroblast Growth Factor 14 (FGF14) Microdeletions

**DOI:** 10.1007/s12311-025-01919-7

**Published:** 2025-10-16

**Authors:** Elena Conci, Thomas L. Kelly, Ruth Armstrong, Pooja Harijan, Rita Horvath

**Affiliations:** 1 Department of Clinical Neurosciences, University of Cambridge, Cambridge, UK; 2 Department of Neurology, Cambridge University Hospitals NHS Foundation Trust, Cambridge, UK; 3 School of Clinical Medicine, University of Cambridge, Cambridge, UK; 4 Department of Paediatric Neurology, Cambridge University Hospitals NHS Foundation Trust, Cambridge, UK; 5 Department of Clinical Genetics, Cambridge University Hospitals NHS Foundation Trust, Cambridge, UK; 6 Department of Clinical Neurosciences, John van Geest Centre for Brain Repair, Forvie Site, Robinson Way, Cambridge, CB2 0PY UK

**Keywords:** Spinocerebellar ataxia 27A, Episodic ataxia, FGF14, Microdeletion, Case report

## Abstract

Spinocerebellar ataxia 27 A (SCA27A) is a form of progressive cerebellar ataxia due to pathogenic variants in the Fibroblast Growth Factor 14 (*FGF14*) gene. The objective of this paper is to characterise the clinical spectrum of SCA27A microdeletions (> 50 bp, <2Mbp), and report two novel cases. Literature searches of PubMed, OMIM and ClinVar were carried out. We identified SCA27A microdeletions in 32 cases across 11 families. The phenotypic presentation is: 75% (24/32) nystagmus, 46% (15/32) ataxia, 21% (7/32) episodic ataxia, 21% (7/32) tremor, 15% (5/32) dysarthria, 34% (11/32) learning disability, 28% (8/32) neuropsychiatric disease. The presentation is variable within and between families. Episodic symptoms, nystagmus, learning disability and neuropsychiatric symptoms occur at an earlier age. Patient 1 represents the first case with a 58 kb *FGF14* deletion who presented with a paroxysmal movement disorder. Patient 2 carries a 545 kb deletion and developed episodic ataxia and trigeminal neuralgia, a novel feature not previously described in this cohort. We report two cases of heterozygous *FGF14* microdeletions: Patient 1 (58 kb) and Patient 2 (545 kb), expanding the phenotypic spectrum of *FGF14* structural variants to 32 cases across 11 families. We review potential mechanism from pre-clinical studies relating *FGF14* haploinsufficiency to cerebellar, cognitive, neuropsychiatric symptoms, as well as trigeminal neuralgia. We propose the hypothesis that the episodic symptoms in SCA27A align with the molecular pathology of a channelopathy and propose management strategies based on this insight.

**Supplementary Information:**

The online version contains supplementary material available at 10.1007/s12311-025-01919-7.

## Introduction

Spinocerebellar ataxias are a heterogenous group of progressive cerebellar ataxias with genetic aetiologies. Spinocerebellar ataxia 27 (SCA27) is associated with pathogenic variants in *FGF14* which is situated on chromosome 13q33. SCA27A is the result of single nucleotide variants [1, 2] and deletions (Table 1) in the *FGF14* gene, whilst SCA27B (recently described) is caused by intronic pathological GAA expansions [14, 15]. In addition, recently a homozygous *FGF14* frameshift variant has been also reported in a child with autosomal recessive cerebellar ataxia with prominent paroxysmal non-kinesigenic dyskinesia [16]. *FGF14*-deficient mice recapitulated symptoms of ataxia and present with a paroxysmal hyperkinetic movement disorder [17–19]. In this review we will focus on SCA27A.

**Table 1:** Clinical phenotypes organised by decreasing size of deletion. For patient series, the age range is reported, with the number of patients indicated in brackets. The symptoms and signs listed represent the combined neurological findings observed across all patients with each specific deletion with the proportion of affected individuals in brackets. SEN = special educational needs (low IQ, dyslexia, special schooling, etc.). NR = not reported. GEN = gaze-evoked nystagmus. V = vertical. H = horizontal. OKR = weak optokinetic reflex. UN = up-beat nystagmus. DN = down-beat nystagmus. ES = esotropia. OSC = oscillopsia. DS = dysmetric saccades. SS = slow saccades. SP = saccadic pursuit. SWJ = intrusive square wave jerks

Size of deletion (kb)	Age years (number of patients)	Age of Onset	Episodic	Ataxia	Nystagmus	Dysarthria	Tremor	SEN	Neuro psychiatric	Other important* Notable	Ref
599	18–87 (6)	< 1–30	+ (1/6)	+ (3/6)	+ (6/6) H GEN (6/6) V GEN (1/6) H + V OKR (4/6)	NR	+	+ (4/5)	EUPD, psychosis, depression, ADHD, anger outbursts (3/5)	Neonatal cervical dystonia, febrile tremors	Paucar et al., 2020 [3]
545	30	24	+	+	+ GEN	-	-	+	ADHD, autism	Trigeminal neuralgia, dysphonia	Patient 2
441	20	< 1	-	+	NR SS	NR	+	+	NR	Lower limb UMN signs, dysmorphic facies, microcephaly and cerebellar atrophy	Planes et al., 2015 [4]
424	4.5 (2, twins)	15/12	+ (2/2)	+ (2/2)	+ (2/2) GEN	NR	+ (2/2)	+ (2/2)	ADHD (2/2)	Psychomotor delay	Blanco-Barca et al., 2016 [5]
252	5	NR	-	-	NR	+	-	+	ADHD, disruptive behaviour disorder	Phonological disorder	ClinVar SCV000804090.2 [6]
235.5	68 − 49 (2)	58 − 46	+ (1/2)	+ (2/2)	+ (2/2) Case 1: DN, ES Case 2: DN, ES, OSC, DS	+ (1/2)	+ (1/2)	+ (1/2)	ADHD		Hoshina et al., 2023 [7]
201.8	6–66 (3)	2–49	+ (1/3)	+ (3/3)	+ (1/3) Case 1: H + V GEN, DS, SWJ Case 2: “abnormal eye movement”	+ (1/3)	+ (1/3)	-	-		Coebergh et al., 2014 [8]
161	1.5–54 (13)	6/12–40	NR	+ (2/13)	+ (12/13) GPN (12/13) UN (9/13) DN (2/13) SP (6/13) DS (2/13)	+ (1/13)	-	-	BDP, depression (1/13)	Seizures	Harris et al., 1993 [9]; Reggae et al., 2003 [10]; Ceroni et al., 2023 (family 2) [11]
97	4.5	< 1	-	+	NR	+	+	+	NR	Dysmorphic facies, acrocephaly	Tucker et al., 2013 [12]
58	14 months	N/A	+	-	-	-	-	-	-	Plagiocephaly, pseudoesotropia	Patient 1
48	10	NR	NR	+	NR	NR	NR	NR	NR	Segmental dystonia Myoclonus	Zech et al., 2021 [13]

**Fig. 1:**
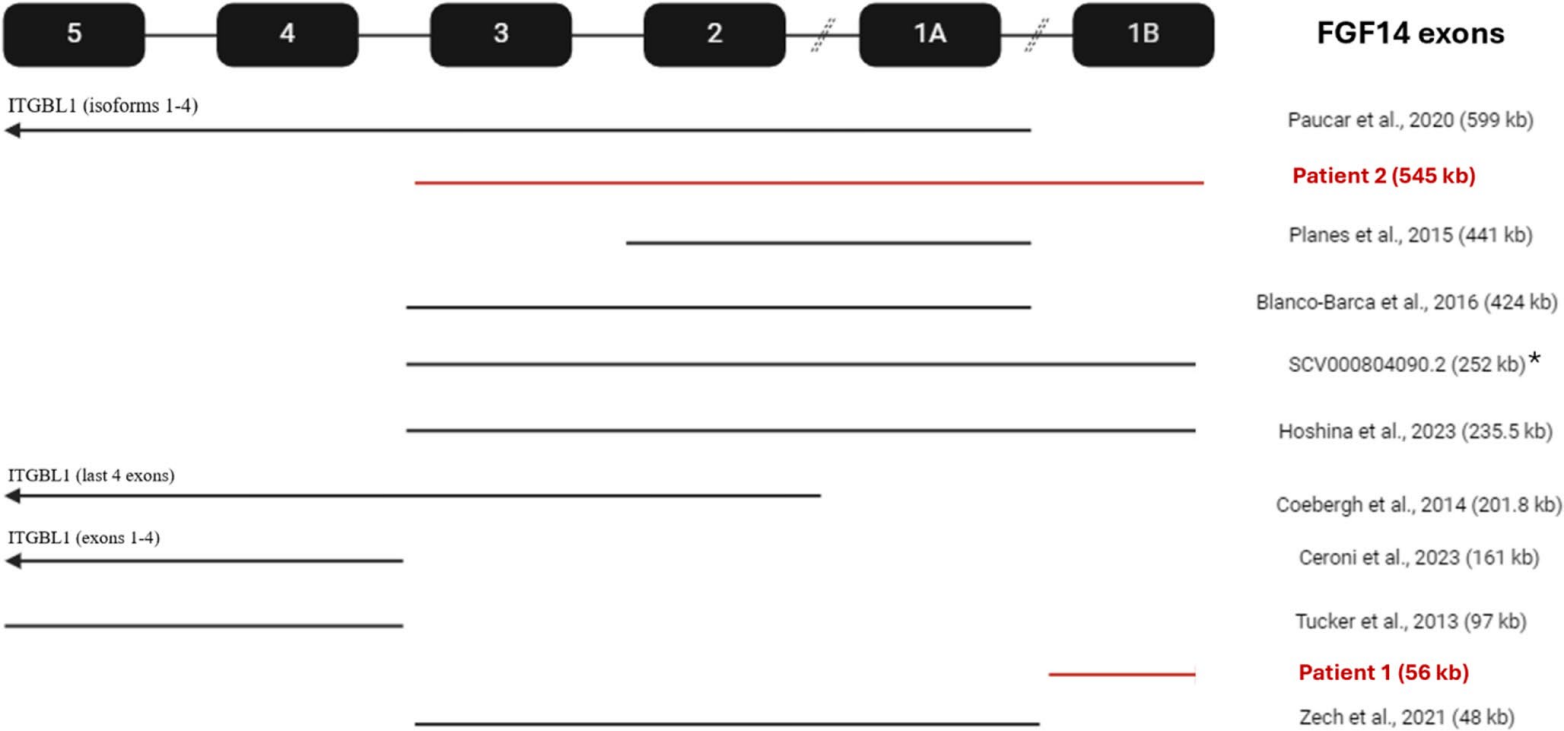
Structural variants in ***FGF14*** gene reported in the literature, compared with variants identified in probands from case series *Two additional CNV were also identified in this patient, one that is pathogenic and one of uncertain significance

The classic clinical findings in SCA27A are cerebellar ataxia, present in >80% of genetically diagnosed patients, including limb and gait ataxia, nystagmus, dysarthria, and tremor, with variable cerebellar atrophy [20]. It is characterised by an early age of onset, including infant-onset, and >50% exhibit intellectual impairment and behavioural issues [20]. In contrast, SCA27B is late onset (median 60-year-old), with 50% of patients having episodic oculomotor (downbeat or horizontal gaze-evoked nystagmus) or limb ataxia 2–4 years before the onset of progressive ataxia with cerebellar atrophy, without intellectual impairment or neuropsychiatric symptoms [21]. Whilst spinocerebellar ataxia is the most typical clinical phenotype of *FGF14* pathogenic variants, some patients show episodic symptoms leading to alternate classifications of episodic ataxia [22, 23] or paroxysmal non-kinesigenic dyskinesia [16, 24]. Additionally, whilst most reported cases of SCA27A are due to single nucleotide variants [1, 2, 22, 25, 26], there have also been reports of microdeletions (Fig. 1), and chromosomal translocations [24] affecting the *FGF14* locus. Comparison of cases linked to structural variants (indels >50 bp) of variable sizes offers insight into how molecular genetics affect the clinical phenotype of SCA27A. We report the phenotype of two patients with structural microdeletions of *FGF14*, in the context of 30 other cases described in the literature (Fig. 1; Table 1).

## Methods

Literature searches of PubMed, OMIM and ClinVar were carried out in June 2024 and repeated in January 2025, including all dates of publications with the search terms “FGF14”, “Fibroblast Growth Factor 14”, “FGF14 deletion”, “SCA27”, “Spinocerebellar ataxia 27”, “SCA27A”, “FGF14 and spinocerebellar ataxia” (Table 2). We excluded genetically confirmed cases of SCA27A due to repeat expansions, single-nucleotide polymorphisms, translocations, deletions >2 Mb or indels at single nucleotide level causing frameshift or truncating mutations. We excluded Chen 2012 as the deletion was too small (10 bp) to draw conclusions about structural variants (>50 bp). Final articles for review after removing duplicates were 7. Two further cases were identified through search of OMIM [12] and ClinVar [6].">

**Table 2:** Result of search from articles using pubmed using various combinations of key words

Key words	Total	Included	Excluded
FGF14	246	5	241
Fibroblast Growth Factor 14	191	7	184
FGF14 deletion	22	7	15
SCA27	27	4	23
Spinocerebellar Ataxia 27	27	7	20
SCA27A	30	7	23
FGF14 AND Spinocerebellar Ataxia 27	63	7	56

## Results

Genetic coordinates (Fig. 1) and clinical details (Table 1) for 11 families with structural variants affecting *FGF14*.

### Patient 1

A 14-month-old male presented with spontaneous movements including bilateral trunk and upper limb twitching several times a day sometimes followed by brief episodes (seconds) of the eyes rolling up and then horizontally, without EEG correlate (supplied in Supplementary material). He was also noted to be fussy about food and had emotional outbursts. He was born following uneventful pregnancy and Caesarean section due to macrosomia. Developmental milestones were normal: he was walking independently at 12 months and started to say “mum” at 14 months. There was no family history of movement disorders; parents were healthy, non-consanguineous, and a three-year-old sister was well.

He had right sided plagiocephaly and right pseudoesotropia due to a broad nasal bridge. Neurological examination was unremarkable, with normal muscle tone, power, reflexes and sensation. Gait was slightly wide based as expected for his age, but he did not appear ataxic. There were no abnormal movements. However, his sitting balance was poor for his age, and he was easily distressed.

He had normal brain and spine MRIs (other than a Rathke cyst), and EEG. Microarray (carried out in the neonatal period as part of investigation for abnormal movements) identified a 58 kb deletion leading to copy number loss of chr13q33.1 (13q33.1(101887591_101944025)x1) which includes the *FGF14* gene (loss-of-function of which is associated with SCA27A) and a paternally inherited copy number loss of chr4q22.3 (4q22.3(94693127_95148910)x1 pat 4q22.3PAT8Q24.13) of uncertain clinical significance.

The child presented to paediatrics; speech and language therapy due to feeding difficulties; occupational therapy and physiotherapy to support trunk strength development; orthotics for excessive passive range of motion of ankles; and clinical genetics due to parental concerns about gait, movements and behaviour. As the episodes of movements were brief, elusive to diagnostic categorisation, and not affecting function he was not medicated.

### Patient 2

A 30-year-old man, who had normal early development except for an episode viral encephalitis (adenovirus positive) aged 5. He was diagnosed with ADHD (based on Conner’s Rating Scales, Strength and Difficulties Questionnaire) and learning disability at age 9 by Child and Adolescent Psychiatrist and Educational Psychologist, respectively, and he was referred to psychiatry at age 19 due to depression, anxiety and aggressive outburst. His first motor symptom, tremor accompanied by vacant episodes, appeared at age 21, and led to redundancy due to risk of personal injury in his manual occupation at a retail warehouse. At age 24, he developed swelling, erythema and tenderness in the maxilla with short-lasting shooting pains that were both spontaneous and evoked by brushing, chewing and cold air. Despite normal orthopantomography, he was treated with antibiotics for suspected dental infection. However, his symptoms persisted, prompting a visit to the emergency department. There, he exhibited gaze-evoked nystagmus, dysmetria, ataxia, dysarthria and dysphonia, raising concern for a stroke. MRI/MRA were unremarkable, and he was diagnosed with trigeminal neuralgia. Gabapentin was trialled with limited success, and his symptoms resolved spontaneously.

At age 27, he experienced a recurrence of trigeminal neuralgia associated with dysphonia and ataxia. Carbamazepine improved his neuralgia but worsened the ataxia, prompting a switch to topiramate. He also experienced an episode of loss of consciousness, which was extensively investigated for central causes, but none was found. Flexible nasendoscopy showed normal vocal cord movement. Given the persistent dysphonia and nystagmus, genetic testing was conducted, revealing a deletion in *FGF14* gene (exons 1–3), confirming a diagnosis of Spinocerebellar Ataxia 27 A (ARRAY ISCN: arr[GRCh38] 13q33.1(101732949_102278224)x1). The episode again resolved spontaneously.

At age 29, he had a third episode of trigeminal neuralgia with dysphonia and ataxia. Examination revealed allodynia without hypesthesia of right trigeminal mandibular distribution with seconds-long electric-shock pains that were both spontaneous and evoked by brushing, chewing and cold air. He also had dysphonia, dysarthria, horizontal and vertical gaze-evoked nystagmus, dysmetria and ataxia, with brisk deep tendon reflexes and normal power and tone. Carbamazepine was reintroduced but resulted in worsening of cerebellar symptoms to the point of wheelchair dependence, as well aggression with ballistic movements. Carbamazepine was discontinued, and neurosurgical consultation was sought for consideration of ablative interventions. Brain MRI showed no trigeminal nerve compression or cerebellar atrophy. His trigeminal neuralgia subsided after several months of using cannabinoid oil. On follow-up, his trigeminal neuralgia, dysphonia, dysarthria, nystagmus, dysmetria, and ataxia had fully resolved.

With regards to his family history, both his children have been diagnosed with ADHD-autism spectrum disorder at a similar age to him (6 and 9 years old). His two brothers and one sister have also inherited the deletion. We have not seen them at our clinic, but they were reported to be unaffected. We have no knowledge of the health of the biological parents, as the patient was raised in foster care.

## Discussion

Here we describe two patients with microdeletions (< 2 Mbp) in *FGF14* gene, leading to either adult-onset episodic ataxia, trigeminal neuralgia, and ADHD-autism spectrum disorder or to an infantile episodic movement disorder. These cases highlight, that SCA27A can be episodic, and expand the phenotypic spectrum of *FGF14* structural variants to include trigeminal neuralgia. In Patient 1, the microdeletion specifically impacts the FGF14b splice variant, which will be crucial in determining whether distinct phenotypes are associated with different FGF14 isoforms.

### Summary of Clinical Presentation of SCA27A Deletion Cases from Literature

Cerebellar features with gait and limb ataxia, nystagmus (gaze-evoked or vertical) and dysarthria are reported in variable proportion in all cases of *FGF14* deletion [3–5, 7, 8, 11–13]: 75% (24/32) have nystagmus, 46% (15/32) ataxia, of which half (7/32, 21%) are episodic. Moreover, 21% (7/32) have tremor, whether postural or intention, and only 15% (5/32) have dysarthria. Episodic ataxia has been reported to be fever induced [3, 5, 8], lasting 4–5 days [5], and to be progressive in frequency and duration with age [7]. Ataxia can lead to progressive cerebellar atrophy [4]. Ataxia can be mild or absent despite deletion of exon 4 and 5 in *FGF14* [11, 12], while deletion in exons 1–3 did result in ataxia, suggesting some genotype-phenotype correlations in SCA27A. Myoclonus and childhood-onset segmental dystonia [13] or other abnormal movements seen in Patient 1 have been also reported.

Intellectual disability and developmental delay have been reported in several cases of *FGF14* deletions, with almost complete penetrance (90%). Notably, in [3], in a Swedish family carrying a 600 kb deletion affecting the whole *FGF14* gene as well as *ITGBL1* gene, all carriers had intellectual disability (II:5 IQ 92, III:1 IQ 65, III:2 IQ 71, IV:1 IQ 72–82), suggesting complete penetrance of this phenotype with *FGF14* allelic loss. Functional studies moreover correlated this with hypometabolism in prefrontal cortex, temporal cortex and cerebellum, strengthening the theory that *FGF14* haploinsufficiency negatively affects neuronal development. Deletions affecting *FGF14* exons 1–3 are consistently associated with cognitive impairment, as evidenced by multiple cases of intellectual disability and progressive cognitive decline, including in the wider family [5–7]. Deletions spanning exons 1–2 are sufficient to produce cognitive deficits, as demonstrated by moderate intellectual disability, speech delay, and microcephaly in the 20-year-old proband [7]. Exon 4 deletions also lead to cognitive impairment, with low IQ, speech delay, and special education needs [12, 27]. However, exceptions were reported [8, 11], where a 200 kbp deletion spanning *FGF14* exons 2–5 and *ITGBL1* gene and a 168 kbp deletion spanning *FGF14* exons 4–5 and ITGBL1 gene, respectively, did not cause cognitive impairment. However, isolated *FGF14* exon 4–5 deletion [12] is sufficient to cause intellectual disability. The reason for this phenotypic variability remains unclear.

Neuropsychiatric features are also prominent in *FGF14* exon 1–3 deletion carriers. ADHD is reported in Patient 2, his son, and other cases [3, 6, 7], often co-existing with aggressive or disruptive behaviour [3, 6]. ADHD or behavioural disorders may precede the onset of motor symptoms in SCA27A. Other neuropsychiatric disorders, such as depression and psychosis, have been documented in carriers [3, 11]. In summary, we may conclude that larger deletions and deletions of exons 1–3 may cause more severe cognitive and neuropsychiatric symptoms.

### Novel Presentation in our Patients

Heterozygous deletion of *FGF14* gene has been repeatedly reported in association with febrile illness [3, 5, 8]. Patient 2 appears to have episodic ataxia not only linked to febrile episodes, but also with trigeminal neuralgia, a state of inflammation of the trigeminal nerve. It is possible that his childhood infectious encephalitis may have been accompanied by febrile episodic ataxia. Episodic ataxia has also been associated with systemic autoimmunity (antiphospholipid syndrome), CSF pleocytosis, type-1 diabetes and hypothyroidism [7].

Episodic dysphonia has been noticed in Patient 2, as well as in two other reported cases [6, 7]. In all these three instances, there is heterozygous deletion in *FGF14* exons 1–3, involving both isoforms FGF14a and FGF14b. It is possible, that *FGF14* haploinsufficiency may affect the firing patterns of vagus nerve in these patients.

In Patient 2, we report the first occurrence of idiopathic trigeminal neuralgia (TN) coexisting with episodic ataxia, nystagmus, dysarthria and dysphonia. The TN diagnosis is well-established, and no secondary pathology was found to explain the condition.

Patient 1 demonstrates episodic body and eye movements, which cannot yet be classified as either ataxia or nystagmus. However, since the microdeletion only encompasses the exon 1b, in theory, this may lead to phenotypic rescue. Patient 1 is the first to define the functional effects of isolated loss of FGF14b splice variant (exons 2–5 unable to join exon 1b due to its loss), where the FGF14a splice variant (exons 2–5 joining exon 1a) may still be active and functional.

### Molecular Data on Channels

The *FGF14* gene is ∼600 kb with five exons, that produce alternatively spliced transcript variants that differ in exon one. The two mRNA transcripts encode protein isoforms of fibroblast growth factor 14: FGF14a (247 amino acids) and FGF14b (252 amino acids) respectively. Fibroblast growth factor 14 (also known as fibroblast homologous factor 4) is part of the family of intracellular FGF [28] that binds to the C-terminus of voltage-gated Na + channels [29–32] and promotes their localization at the action potential initiation site (AIS) of neurons [33–35] providing fine-tuned regulation of their neuronal firing [36]. The two FGF14 isoforms have been shown to interact differentially across NaV subtypes [37–40] although this finding is yet to be validated in humans. We propose that the pathophysiology of *FGF14* microdeletions mirrors that of truncating single nucleotide variant, both leading to *FGF14* haploinsufficiency and resulting in disrupted regulation of voltage-gated sodium channel dynamics in neurons [41].

Preclinical evidence strongly links *FGF14* homozygous deficiency to cerebellar dysfunction in mouse models. *FGF14*-deficient (homozygous knock-out) mice exhibit ataxia [19] and paroxysmal dyskinesias [17], which are associated with reduced NaV1.6 expression at the axonal initial segment and impaired Purkinje cell excitability [35, 42–44]. Restoration of *FGF14* expression in homozygous knockout mice via viral delivery [45] reversed ataxia and normalized Purkinje cell firing, demonstrating a causal link.

Preclinical studies show that FGF14 is crucial for synaptic plasticity and neurogenesis. *FGF14* homozygous knockout mice exhibit abnormal synaptic plasticity [46], reduced neurogenesis [47] in the hippocampus, and impaired spatial learning [18], linking *FGF14* loss to cognitive impairment in the SCA27 phenotype. While preclinical studies linking *FGF14* haploinsufficiency to ADHD are limited, there is evidence associating *FGF14* with autism and schizophrenia [48–51].

We hypothesize that *FGF14* haploinsufficiency predisposes individuals to TN following injury or inflammation Supporting this, FGF14 regulates voltage-gated sodium channels, including NaV1.3 and NaV1.6, in neuronal tissues [37, 52, 53]. Genetic variants associated with TN have been identified in NaV1.3 [54] and NaV 1.6 [55]. Moreover, upregulation of NaV1.3 has been linked to TN in both human studies [56] and animal models [57–60]. Loss of FGF14 may lead to abnormal NaV1.3 activity, amplifying repetitive firing in the trigeminal nerve and contributing to central sensitization. Further studies are needed to confirm this. While there is also evidence of NaV1.6 mediating central sensitization in neuropathic pain models [61, 62], studies are needed to confirm the effect of FGF14 on NaV1.6 in the trigeminal nerve.

### Treatment

Treatments may be recommended based on the emerging insight that the molecular pathology of SCA27A is of a sodium channelopathy and contributes to the phenotype of episodic ataxia 9 [23]. The episodic nature of TN, characterized by brief spasms, may be linked to the loss of long-term inactivation in NaV1.3 or NaV1.6. Carbamazepine and lamotrigine are effective in trigeminal neuralgia [63] because they shift the steady-state inactivation of NaV channels, including NaV1.3 and NaV1.6, to more hyperpolarized potentials [64], counteracting the loss of long-term inactivation [65] in the trigeminal nerve. While carbamazepine was effective for the TN, it worsened Patient 2’s movement disorder. Lamotrigine may be an alternative, as it has shown efficacy in other sodium channelopathies like paramyotonia congenita [66]. Additionally, acetazolamide, which has shown efficacy in improving ataxia in SCA27A [7], SCA6 [67], hereditary paroxysmal ataxia [68, 69], and episodic ataxias [70], could also be trialled empirically.

Our patient self-medicated with Cannabidiol oil and reported benefit in the control of his TN. Cannabidiol is the non-psychoactive component of *Cannabis sativa* and it has been shown in-vitro to non-selectively reduce the peak inward conductance of NaV1.1–1.7.7 channels by shifting the voltage-dependence of inactivation to more hyperpolarised states [71] and stabilising their inactive state in a temperature-dependent manner [72]. CBD reduces the frequency and severity of temperature induced seizures and improves autistic-like social behaviours in the in-vivo mouse model of Dravet syndrome (SCN1A+/-) [73]. This has been corroborated in clinical trials that also demonstrate a reduction in seizure frequency with CBD in patients with epileptic sodium channelopathies, such as Dravet [74] and Lennox-Gestaut [75] syndrome.

### Limitations

The limitations to this study include lack video recording of movement semeiology, and lack of standardised IQ scores for both patients. Moreover, Patient 2’s neuropsychiatric diagnoses by the Child and Adolescent Psychiatrist have not been confirmed with more rigorous assessments or revised. The initial assessment was strongly in favour of a diagnosis of ADHD, but there is also a suggestion of autism-spectrum-disorder that was at that stage uncertain and required further follow up for confirmation. We do not have further documentation regarding whether this follow-up took place. Moreover, it is possible that publication bias has limited the number of detailed descriptions of infantile cases. This makes comparison with Patient 1’s presentation challenging.

## Conclusions

We characterized the phenotypic presentation of SCA27A resulting from *FGF14* deletions in 32 published cases across 11 families, and report two new cases, further expanding the clinical spectrum. The clinical presentation is variable within and between families, but some patterns emerge: episodic symptoms, nystagmus, learning disability and neuropsychiatric symptoms occurring at an earlier age, but similar in patients of different ethnic origin. Finally, preclinical findings suggest that altered sodium channel function may underlie the clinical symptoms, pointing to potential novel treatment options for patients with SCA27A .

## Supplementary Information

Below is the link to the electronic supplementary material.

Supplementary Material 1

## Data Availability

No datasets were generated or analysed during the current study.
