# Supplementary material for: Spinocerebellar Ataxia 27 A with Episodic Ataxia: Case Series of Fibroblast Growth Factor 14 (FGF14) Microdeletions
Source: Cerebellum. 2025 Oct 16;24(6):166. doi: 10.1007/s12311-025-01919-7 (PMC12532728; doi:10.1007/s12311-025-01919-7)
Supplement: Supplementary file 1 — Supplementary Material 1 [file 12311_2025_1919_MOESM1_ESM.docx]

Supplementary Material

The following is an excerpt from the EEG report of patient 1 describing the EEG findings during one ‘episode’ of movement which confirmed no concurrent focal epileptiform discharges. Provided with thanks from colleagues in the neurophysiology department.

“1 typical episode was captured during the recording, Mum describes, left arm twitching and eye rolling, lasting < 1 minute. This occurred around […] – Мum pressed the event marker.

Eye closure elicits a symmetrical posterior rhythm at 5–6 Hz (up to 250 µV).

Background activities consist of widespread theta activity at 4-7.5 Hz (up to 90 µV), mixed with underlying, post-central delta activity at 1-3.5 Hz (up to 115 µV) and superimposed, lower voltage, widely distributed beta activity at 14-30 Hz (< 40 µV). Lambda waves are present.

Prominent, rhythmic 4-5 Hz (up to 125 µV) theta range activity is intermixed with the background delta/theta activities, maximal over the central and posterior areas. In sleep, vertex sharp waves, sleep spindles, POSTS and K-complexes are seen. As sleep deepens, diffuse slow/delta activity (up to 450 μV), dominates the recording.

Upon EEG review, it is seen that [Patient 1] frequently alerts during sleep, with movement and arousal seen on the EEG recording. In addition, the bilateral, Deltoid EMG leads, show associated upper limb movements at the time of alerting. No clear EEG correlates occur.

Episodes:

During the reported episode of left arm twitching and eye rolling […] Арart from muscle and movement artefact, there are no obvious changes seen in the on-going/baseline EEG. The EEG shows [Patient 1] moving and alerting from sleep during this time.”
